# Differential Effects of the Mitochondria-Active Tetrapeptide SS-31 (D-Arg-dimethylTyr-Lys-Phe-NH_2_) and Its Peptidase-Targeted Prodrugs in Experimental Acute Kidney Injury

**DOI:** 10.3389/fphar.2019.01209

**Published:** 2019-11-08

**Authors:** Jean-Christophe Wyss, Rajesh Kumar, Josip Mikulic, Manfred Schneider, Jean-Luc Mary, Johannes D. Aebi, Lucienne Juillerat-Jeanneret, Dela Golshayan

**Affiliations:** ^1^Transplantation Center and Transplantation Immunopathology Laboratory, Department of Medicine, Centre Hospitalier Universitaire Vaudois (CHUV) and University of Lausanne (UNIL), Lausanne, Switzerland; ^2^Medicinal Chemistry, Roche Pharma Research and Early Development (pRED), Roche Innovation Center, F. Hoffmann-La Roche Ltd, Basel, Switzerland; ^3^University Institute of Pathology, CHUV and UNIL, Lausanne, Switzerland

**Keywords:** oxidative stress, renin–angiotensin system, aminopeptidase A, experimental kidney disease, acute renal injury

## Abstract

The mitochondria-active tetrapeptide SS-31 can control oxidative tissue damage in kidney diseases. To investigate other potential beneficial nephroprotective effects of SS-31, *in vivo* murine models of acute tubular injury and glomerular damage were developed. Reduction of acute kidney injury was demonstrated in mice treated with SS-31. The expression of mRNAs involved in acute inflammatory and oxidative stress responses in the diseased kidneys confirmed that SS-31 could regulate these pathways in our *in vivo* models. Furthermore, *ex vivo* histoenzymography of mouse kidneys showed that aminopeptidase A (APA), the enzyme involved in the processing of angiotensin (Ang) II to Ang III, was induced in the diseased kidneys, and its activity was inhibited by SS-31. As the renin–angiotensin system (RAS) is a main regulator of kidney functions, the modulation of Ang receptors (ATR) and APA by SS-31 was further investigated using mRNAs extracted from diseased kidneys. Following acute tubular and/or glomerular damage, the expression of the AT_1_R mRNA was upregulated, which could be selectively downregulated upon SS-31 administration to the animals. At the same time, SS-31 was able to increase the expression of the AT_2_R, which may contribute to limit renal damage. Consequently, SS-31-based prodrugs were developed as substrates and/or inhibitors for APA and were screened using cells expressing high levels of APA, showing its selective regulation by α-Glu-SS-31. Thus, a link between SS-31 and the RAS opens new therapeutic implications for SS-31 in kidney diseases.

## Introduction

Within the kidney, oxidative stress injury to glomerular, tubular, or endothelial cells is the initiating cause of many acute and chronic lesions, leading to progressive dysfunction and end-stage renal disease (Lv et al., 2018; Daenen et al., 2019). An oxidative stress may be induced by either metabolic disorders and inflammation, such as hypertension and diabetes, or drug toxicity such as radiology contrast agents, antibiotics, and anticancer chemotherapeutics (Hulse and Rosner, 2019). The main organelle producing reactive oxygen species (ROS) is the mitochondria, but other metabolic pathways may also produce an oxidative stress and induce various stress responses in the kidney. ROS can either directly damage biological structures or indirectly activate cellular signaling pathways that are deleterious to the normal function of organs. Reducing ROS generation in response to tissue stress may prevent injury to the kidney (Koga et al., 2012). Thus, antioxidants, by scavenging oxidants or by inhibiting oxidative stress pathways, are interesting agents to protect tissues and organs. However, active oxygen species are also fundamental cellular signaling mediators and effectors, necessary for several important physiological processes.

Several antioxidants have been evaluated in experimental models of acute kidney injury and clinical trials, including superoxide dismutase (SOD) mimetics, glutathione, *N*-acetylcysteine, vitamin E, lipoic acid analogs, and, more recently, the cell-permeable mitochondria-active tetrapeptide SS-31 (D-Arg-dimethylTyr-Lys-Phe-NH_2_, Elamipretide/Bendavia) (reviewed in Tabara et al., 2014). SS-31 selectively targets the mitochondrial inner membrane and prevents oxidative stress by enhancing oxidative phosphorylation coupling, thus improving ATP production. SS-31 was shown in several cellular and animal models to protect the kidney against ischemic injury by reducing death of tubular cells and by enhancing the proliferation of surviving tubular cells (Zhao et al., 2004; Thomas et al., 2007; Szeto et al., 2011; Kloner et al., 2012; Szeto, 2014; Eirin et al., 2014; Tabara et al., 2014). SS-31 also controls organ damage mediated by the NF-κB pathway (Mizuguchi et al., 2008; Huang et al., 2013) decreasing the production of pro-inflammatory cytokines (Imig and Ryan, 2013) and the recruitment of macrophages and neutrophils (Szeto et al., 2011). However, cellular pathways other than direct anti-oxidative and inflammatory responses may be involved in the beneficial effects of SS-31.

Our main aims in the present report were to determine which beneficial effect SS-31 has on stress responses leading to glomerular and tubular injuries, and consequently to design targeted tissue-selective analogs of SS-31 as prodrugs. We therefore first evaluated the protective effect of SS-31 in two murine models of kidney diseases: acute tubulo-interstitial injury following aristolochic acid (AA) administration and adriamycin (ADR)-induced progressive glomerular damage. Using these experimental models, we determined the level of induction of oxidative stress-related molecules, inflammatory mediators, and cell proliferation pathways in the injured kidneys, in the absence or presence of SS-31. We also studied the modulation of the renin–angiotensin system (RAS), a major mediator of renal diseases when overactivated (Zhong et al., 2017). In the past years, we have investigated the therapeutic potential of specific drug-targeting strategies (Berger et al., 2000; Berger et al., 2003). More recently, we have designed functionalized inhibitors of the cell membrane-inserted enzyme γ-secretase (a component of the Notch signaling pathway) as substrates for the cell-surface peptidase γ-glutamyltranspeptidase (γ-GT) that is upregulated in acutely injured renal tubular cells. These prodrugs were shown to specifically enhance exposure of the kidneys to the therapeutic inhibitors (Juillerat-Jeanneret et al., 2015; Wyss et al., 2018). Based on these previous results, we have designed and evaluated prodrugs of SS-31 as potential substrates and/or inhibitors of aminopeptidase A (APA), a peptidase also overexpressed in rodent and human injured kidneys (Song et al., 1994; Troyanovskaya et al., 1996; Wolf et al., 2000).

## Materials and Methods

### Synthesis Procedures

Mass spectra were recorded on an SSQ7000 (Finnigan-MAT) spectrometer for electron impact ionization. Liquid chromatography–mass spectrometry (LC/MS) was recorded on Agilent 1290 LC with CTC PAL coupled to Agilent 6520 QTOF. High-pressure liquid chromatography (HPLC) was performed on column: symmetry C18, 5 µm, 250 × 4.6 mm, and 100 Å; buffer A: 0.1% trifluoroacetic acid (TFA) in water; buffer B: 0.1% TFA in CAN; gradient: (depending of peptide sequence) in 30 min at 1 ml/min; temperature: 25°C; and detector: 210 nm.

### Preparation of SS-31 and Analogs

All peptides were purchased from Polypeptide France and were prepared by manual solid phase peptide synthesis using the 9-fluorenylmethyloxycarbonyl (Fmoc) strategy. The C-terminal phenylalanyl residue was coupled to the resin (p-methylbenzhydrylamine polystyrene–1% divinylbenzene)* via *a Rink amide linker [p-(Fmoc-2,4-dimethoxybenzyl)-phenoxyacetic acid]. The other amino acid residues were incorporated by a succession of Fmoc deprotection and amino acid coupling cycles. After solid-phase assembling of the peptide, the cleavage reaction from the resin and a concomitant side-chain deprotection in one step with TFA yielded directly the crude peptide with C-terminal amides. The crude preparation was precipitated and dried. Purification was performed by preparative reverse-phase HPLC in the TFA buffer. Each purified peptide obtained was tested by ion pair chromatography–HPLC against the specification before entering the final lot. The selected pools were mixed to form a homogeneous solution in water before freeze-drying and packaging.

***SS-31 (D-Arg-DMT-Lys-Phe-NH*** ***_2_*** ***.3TFA)*** was synthesized by solid-phase peptide synthesis as described above using Fmoc-L-Phe, Fmoc-L-Lys(BOC)-OH, (S)-Fmoc-2,6-dimethyltyrosine, and Fmoc-D-Arg(Pbf)-OH to give the final peptide. MS: 640.39 (M + H)^+^.

***α-Glu-SS-31 (H-Glu-D-Arg-DMT-Lys-Phe-NH*** ***_2_*** ***.2TFA)*** was synthesized by solid-phase peptide synthesis as described above using Fmoc-L-Phe, Fmoc-L-Lys(BOC)-OH, (S)-Fmoc-2,6-dimethyltyrosine, Fmoc-D-Arg(Pbf)-OH, and Fmoc-L-Glu(OtBu)-OH.H_2_O to give the final peptide. MS: 769.44 (M + H)^+^.

***γ-Glu-SS-31 (H-γ-Glu-D-Arg-DMT-Lys-Phe-NH*** ***_2_*** ***.2TFA)*** was synthesized by solid-phase peptide synthesis as described above using Fmoc-L-Phe, Fmoc-L-Lys(BOC)-OH, (S)-Fmoc-2,6-dimethyltyrosine, Fmoc-D-Arg(Pbf)-OH, and Fmoc-L-Glu(OH)-OtBu to give the final peptide. MS: 769.66 (M + H)^+^.

### Animal Models of Induced Kidney Diseases

All experiments were conducted in accordance with federal and local regulations, according to a protocol approved by the animal ethics committee of the Canton de Vaud, Switzerland (No. 2655.0). Kidney injury was induced by intraperitoneal (i.p.) injection of AA (Sigma-Aldrich, 1 × 5 mg/kg) or ADR (Adriblastin, Pfizer, 1 × 10 mg/kg) in 10-week-old BALB/c male mice (n = 5–7 mice/experimental group). SS-31 analogs were diluted in 0.9% NaCl and administered i.p. once a day, starting 1 day before the disease-inducing drugs (day −1) at a dose of 3 mg/kg and then daily until day 6. The animals were weighted at days 0, 3, and 6, and sacrificed at day 7. The level of protein in urine was semi-quantitatively assessed using Albustix reagent strips (Bayer, Basel, Switzerland). At the end of the treatment period, the mice were sacrificed to remove both kidneys. The kidneys were spliced in four equal fragments containing the cortex and medulla. One fragment was immediately snap-frozen in liquid nitrogen for real-time quantitative polymerase chain reaction (qRT-PCR) and Western blot experiments, one fragment was included in OCT (Tissue-Tek, VWR International, Dietikon, Switzerland) and frozen for histoenzymography, one fragment was frozen at −80°C and used for pharmacokinetic (PK) measurements, and one fragment was fixed in 4% paraformaldehyde and included in paraffin for histology. Hematoxylin/eosin and Masson’s trichrome blue stainings of paraffin-embedded mouse kidney sections were performed using standard routine procedures to evaluate the level of kidney damage.

### HPLC Procedures and PK of SS-31 Analogs in Mouse Plasma, Liver, and Kidney

For PK evaluation, SS-31 was administered i.p. to male mice in suspension (gelatine/saline 7.5%/0.62% in water) using an administration volume of 4 ml/kg. Blood samples were collected in tubes containing EDTA as an anticoagulant, and plasma was separated by centrifugation and stored at −80°C. Liver and kidney samples (∼100 mg aliquots) were homogenized in three volumes of water using Precellys tissue homogenization tubes (precellys.com). Twenty-five microliters of each tissue homogenate was further diluted with 25 µl blank plasma to produce the final tissue homogenate for extraction. Prepared samples were stored at −20°C before analysis.

All samples were analyzed using protein precipitation followed by LC-MS/MS analysis. Briefly, 50 µl plasma or final tissue homogenate was mixed with 50 µl 0.5 M HClO_4_/acetonitrile 9/1 (containing 200 ng/ml bosentan as an internal standard). Samples were stirred and 300 µl water was added, followed by centrifugation at 5600 rpm (4°C, 10 min). Ten microliters of supernatant was analyzed by LC/MS. Calibration standards in plasma were prepared the same way. The LC columns and conditions used were as follows: Phenomenex, Polar RP, 4 µm, and 50 × 2.1 mm at 0.4-ml/min flow rate with a 3-min gradient from 95% solvent A to 95% solvent B (solvent A: water/acetonitrile/HCOOH, 90:10:0.1 with 10 mM NH_4_ formate; solvent B: water/ACN/HCOOH, 10:90:0.1 with 10 mM NH_4_ formate). Mass spectrometric conditions were as follows: Thermo TSQ Vantage with positive heated electrospray ionization in MS/MS mode. Mass transitions of the compounds were *m*/*z* 320.7 to 119.9. Relative concentrations of the drug and metabolites were established by the percentage of peak area ratio compared to time zero spiked compounds. PK parameters for all studies were calculated using Phoenix WinNonlin Software.

### Histoenzymography

Enzymatic activities were evaluated by histoenzymography on OCT-embedded frozen kidneys sections (7 µm), as previously described (Juillerat-Jeanneret et al., 1992; Juillerat-Jeanneret et al., 2000; Juillerat-Jeanneret et al., 2003). Briefly, slides were fixed for 5 min in cold (−20°C) acetone, air dried at room temperature, rehydrated for 5 min in 0.9% NaCl, and exposed to the α-Glu-4-methoxy-β-naphtylamide (APA; Bachem) or γ-Glu-4-methoxy-β-naphtylamide (γ-GT; Serva) substrates and Fast Blue B (Sigma-Aldrich) at 37°C for 15 to 60 min until a red coloration was visible. The slides were washed in distilled water, counterstained with hematoxylin (Mayer) for 1 min, rinsed under tap water and then with Scott solution for 1 min, and mounted in Aquamount (Immu-mount; Thermo Shandon, Pittsburgh, PA, USA) and analyzed with a Nikon Eclipse E800 microscope and digital DXM1200 camera using ACT-1 software.

### Real-Time Quantitative PCR

Total RNA was extracted from frozen kidney fragments of either untreated mice or mice treated with the various drugs (n = 5–7 mice per experimental group) using the TRIzol reagent (Life Technologies, USA) as per the manufacturer’s instructions. Briefly, 10 mg kidney sample was homogenized using a polytron (VWR International). The nucleic acids were purified by chloroform/isopropanol extraction, quantified with the NanoDrop-ND2000 (Thermo Scientific, USA), and treated by DNase (Promega, USA). DNase-treated RNA samples (260:280-nm absorbance ratio of 1.9–2.0) were subjected to cDNA synthesis with the iScript™ cDNA Synthesis Reverse Transcription (RT) kit (Bio-Rad Laboratories, USA) as per the manufacturer’s instructions. For gene expression profiling, SYBR Green (SensiMixTM SYBR kit, Quantace)-based qPCRs were performed for quantification of a particular transcript using specific primers with Rotor-Gene 6000 instrument (Corbett Research, Australia). Intron spanning and exon-specific primers (sequences provided in **Supplementary Table S1**) were designed and synthesized (Microsynth, Switzerland). Standard curve analysis (>80% efficiency with single melting curve) was performed to validate the primers, and PCR amplicons were checked on ethidium bromide-containing agarose gels. To calculate the relative changes in mRNA expression, the ddC(T) method was used (Livak and Schmittgen, 2001). Gene expression levels were normalized to *Gapdh*, and the control animal group was assigned 100%.

### Cells and Culture Conditions

The preparation and characterization of rat brain-derived EC219 endothelial cells were previously described (Juillerat-Jeanneret et al., 1992). These cells express high levels of APA and γ-GT activities and can be used to measure kinetics in living cells as APA and γ-GT-positive representative cell models. The cells were grown in Dulbecco’s modified Eagle medium (DMEM) containing 1 g/L glucose, 10% heat-inactivated fetal calf serum (FCS), and antibiotics on uncoated cell culture plastic plates and flasks. MDCK and LLCPK cells were obtained from the American Tissue Culture Collection (ATCC, Manassas, VA, USA) and were grown in DMEM containing 4.5 g/L glucose, 10% FCS, and antibiotics. mCCD, and mDCT cells were a kind gift from O. Bonny (UNIL, Lausanne, Switzerland) and were cultured in DMEM (Ham’s F12 1:1 vol/vol, 60 nM sodium selenate, 5 μg/ml transferrin, 50 nM dexamethasone, 1 nM triiodothyronine, 10 ng/ml epidermal growth factor, 5 μg/ml insulin) and 2% FCS. The characteristics of the used cell lines are shown in **Supplementary Table S2**.

### Determination of Enzyme Kinetics in EC219 Cells

Stock solutions of α-Glu-AMC (APA) and γ-Glu-AMC and Gly-Gly (γ-GT) substrates, all from Bachem, were prepared at 10 mM in DMSO. EC219 cells were grown to confluence in 96-well plates (Corning, NY, USA), then the culture medium was removed, and the cell layers washed once with 100 µl PBS and 100 µl of substrates at decreasing concentrations (320–10 µM) of α-Glu-AMC or γ-Glu-AMC and 20 mM Gly-Gly substrates diluted in PBS and increasing concentrations (0–20 µM) of SS-31 analogs were added. The increase in absorbance was measured at 37°C in a thermostated fluorescence multiwell-plate reader (CytoFluor Series 4000; PerSeptive Biosystems, MA, USA) at λ_ex = _360 nm/λ_em = _460 nm using the linear portion of the curves. The kinetics values were then graphically determined or according to Wu and colleagues (Wu et al., 2011).

### Western Blots

EC219 cells were grown in RPMI medium containing 10% FCS and antibiotics until 95% confluence. Then 0.1 µM angiotensin (Ang) II (Bachem), 10 µM losartan (Sigma-Aldrich), and 100 µM SS-31, either alone or in combination, were added for 24 h. The cell layers were washed in PBS and the pellets suspended in the SDS-PAGE protein loading buffer. SDS-PAGE electrophoresis was performed in 10% gels, and the proteins were transferred to a PVDF membrane (Bio-Rad Laboratories). The membranes were probed sequentially with a rabbit monoclonal anti-AT_1_R antibody (clone ab124734, dilution 1/500; Abcam), followed by an anti-rabbit IgG-HRP antibody (dilution 1/1000; Cell Signaling Technology) and Radiance Plus (Azure Biosystems, Dublin, CA). The whole process was repeated on the same membrane with the anti-AT_2_R antibody (Abcam; ab92445, dilution 1/500) and the anti-β-actin antibody (clone 4967, dilution 1/2500; Cell Signaling Technology). Images of chemiluminescence were taken using FUSION-FX (Fisher Biotec). Membranes were analyzed using Photofiltre software and GelAnalyzer software (lazarsoftware).

### Statistical Analysis

Results were averaged for all animals, and means ± SD were calculated. The level of statistical significance between experimental groups was assessed using a paired Student’s *t*-test or one-way analysis of variance along with Tukey’s post-test for multiple comparisons (GraphPad Prism version 6, California). *p* Values < 0.05 were considered significant (*p < 0.05, **p < 0.01, ***p < 0.001).

## Results

### *In Vivo* PK and Nephroprotective Effects of SS-31 in Experimental Acute Kidney Injury

Our first aim in this research was to explore whether SS-31 had beneficial effects on stress responses in acutely injured kidneys other than by controlling the mitochondrial oxidative stress response as previously described (Szeto et al., 2011) and accordingly to design targeted and tissue-selective SS-31 analogs. Using a murine experimental model, we first determined the PK of SS-31 in mouse plasma, liver, and kidney following i.p. administration (**Table 1**). Following a 30-mg/kg injection, the maximal concentration of SS-31 was reached after 1 h and was comparable in the plasma, the liver, and the kidney, but the total concentration AUC_inf_ was slightly higher in the kidney compared to the other organs, which is also reflected by a longer half-life (*T*_1/2_). SS-31 dosage corresponded to a mean kidney concentration (total kidney extract) of SS-31 (MW 639.79 g/mol) of ∼140 nM.

**Table 1:** *In vivo* pharmacokinetics of SS-31 after i.p. administration (30 mg/kg) in male mice; plasma, liver and kidney exposures. (n = 6 mice).

	Plasma	Liver	Kidney
***C*** ***_max_*** *** [ng/ml]***	19992	12695	17236
***T*** ***_max_*** *** [h]***	1.0	1.0	1.0
***AUC*** ***_4h _*** [h*ng/ml]	21120	20709	27801
***AUC*** ***_inf_*** [h*ng/ml]	21138	22101	31280
***T*** ***_1/2 _*** ***[h]***	0.3	0.9	1.1

**Table 2:** Stability of SS-31 and α-Glu-SS-31 in mouse plasma.

Time [h]	Stability [% of T_0_]		Release of SS-31
	SS-31	α-Glu-SS-31	from α-Glu-SS-31 [%]
0	100	100	7.5
0.5	89.3	68.5	9
1	85.3	68.3	9
3	50.1	66.9	10
5	43	65.8	9

SS-31 or α-Glu-SS-31 (at 1 μM concentration) was added to heparinized plasma obtained from healthy wild-type mice and incubated at 37°C. Aliquots were removed at 0, 0.5, 1, 3 and 5 h and analyzed. Stability is expressed as [%] of initial concentration at T0.-

**Figure 1:**
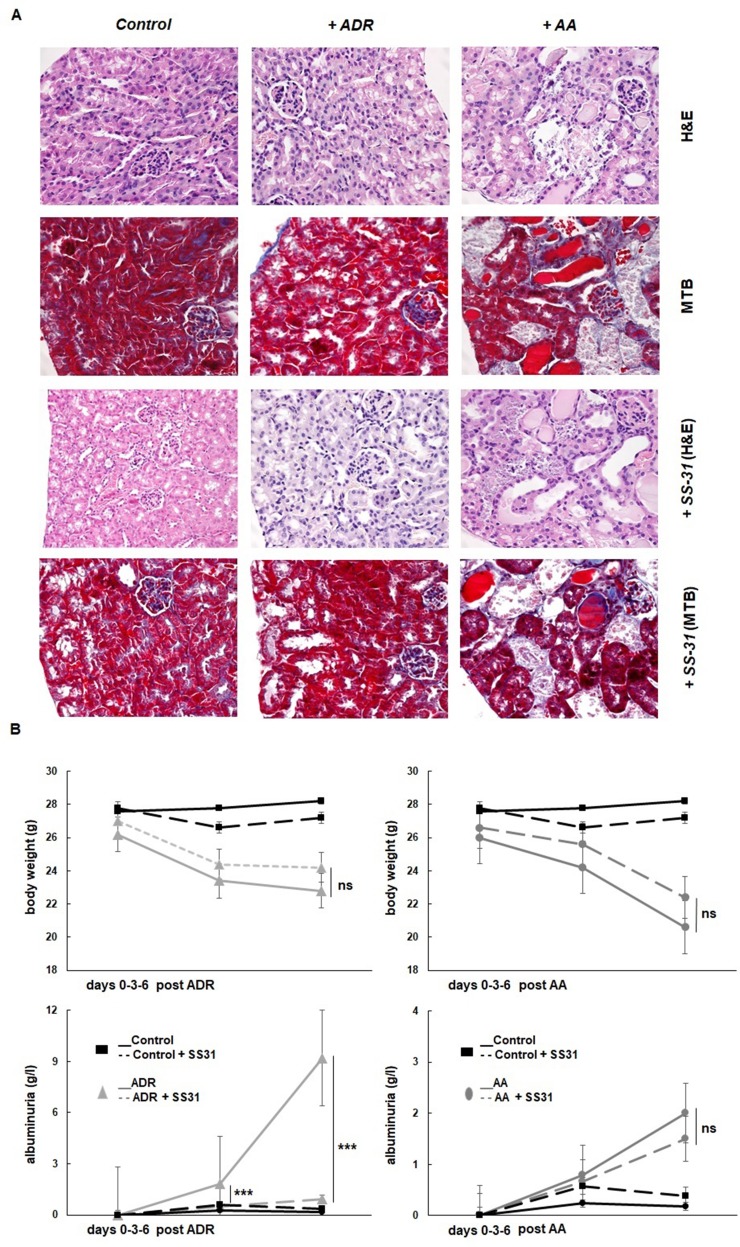
Experimental mouse models of kidney diseases. Kidney injury was induced either by i.p. injection of aristolochic acid (AA, 1x5mg/kg) or adriamycin (ADR, 1x10mg/kg) in 10 weeks old BALB/c male mice (n = 7 animals per experimental group). **(A) **Hematoxylin/eosin (H&E) or Masson’s trichrome blue (MTB) staining of kidney sections of healthy wild-type control mice and mice treated with ADR or AA, without (above and middle panels) or with SS-31 (lower panels). Representative renal cortical sections per experimental groups are shown (200x magnification). **(B)** Renal injury was induced by ADR or AA. SS-31 (3 mg/kg) was administered i.p. once a day, starting one day before the disease-inducing drugs (day −1) and then daily until day 6 (dotted lines) (n = 5 mice per experimental group). The animals were monitored clinically daily, weighted and the level of albuminuria was semi-quantitatively assessed at day 0, day 3 and day 6, and averaged for all animals. Results are presented as means ± sd for all mice in each experimental group, with comparisons between SS-31-treated *versus* non-SS-31-treated diseased animals (***p < 0.001).

The potential nephroprotective effects of SS-31 were evaluated in the *in vivo* murine models of acute renal tubulo-interstitial or glomerular injury, selectively induced by AA or ADR, respectively. The histological evaluation of the organs retrieved from the experimental mice after 1 week confirmed the development of acute kidney damage in the two models, more acutely severe after AA exposure (**Figure 1A**). The administration of AA induced extensive tubulo-interstitial lesions (mainly affecting proximal tubules), resulting in acute kidney insufficiency (mean creatinine 258 ± 92 µmol/L *vs.* 44 ± 5 µmol/L, in the AA and control groups, respectively), rapid weight loss, and death of the animal depending on its severity. **Figure 1A** indeed shows distorted tubular architecture with loss of brush borders in the proximal tubules, focal necrosis, and tubules filled with hyaline casts and cells debris. In comparison, ADR administration results mainly in glomerular podocyte injury and inflammation, which is evidenced clinically by rapid-onset proteinuria but more progressive and late kidney dysfunction following focal glomerular sclerosis (mean creatinine 33 ± 5 µmol/L at the time of sacrifice, in the ADR group). Accordingly, diseased (AA and ADR-treated) and SS-31-treated mice were monitored throughout the experiments for weight and proteinuria (**Figure 1B**). SS-31 administration statistically improved albuminuria in the ADR model of glomerular damage, while in the AA model, SS-31-treated mice were more protected against acute kidney failure and its consequences as reflected by statistically better kidney function (mean creatinine 162 ± 21 µmol/L) and a trend toward less weight loss at the end of the experiment. Kidney histology of mice exposed to either AA or ADR was also better preserved under SS-31 treatment.

### Effects of SS-31 on Induced Stress Responses and Inflammation During Acute Renal Injury

**Figure 2:**
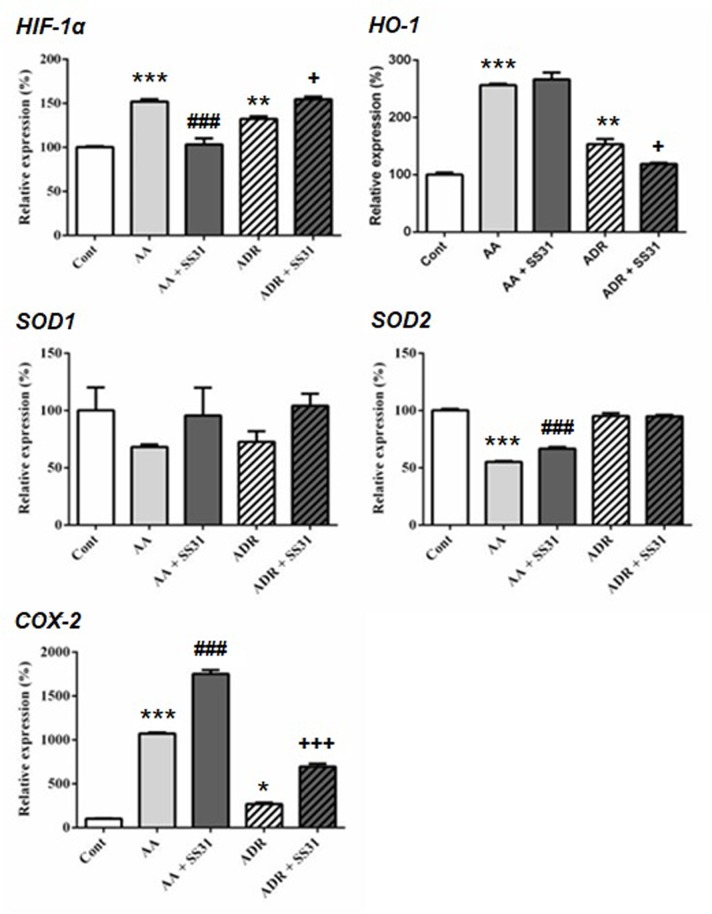
Expression of the oxidative stress pathway-responsive genes in the kidneys of mice exposed to aristolochic acid or adriamycin, without or with SS-31. Acute renal injury was induced by i.p. injection of aristolochic acid (AA) or adriamycin (ADR) in 10 weeks old BALB/c male mice (n = 5 mice per experimental group), without or with the administration of SS-31 (AA + SS31 or ADR + SS31). At the end of the experiment (day 7), the animals were sacrificed and mRNAs were extracted from the snap-frozen kidneys. Healthy wild-type BALB/c male mice were used as controls (Cont). The levels of expression of the mRNAs for hypoxia inducible factor1-α (HIF-1α), heme oxygenase-1 (HO-1), superoxide dismutase-1 (SOD1), SOD2 and cyclooxygenase-2 (COX-2) were quantified by qRT-PCR. Results were averaged for all animals in each experimental group and are presented as % of changes in the mRNA levels in the treated animals *versus* control animals ± sem. *: AA or ADR compared to Cont; ^#^: AA + SS-31 or ^+^: ADR + SS-31 compared to AA or ADR alone, respectively. (*p < 0.05; **p < 0.01; ***p < 0.001; applies to all symbols).

**Figure 3:**
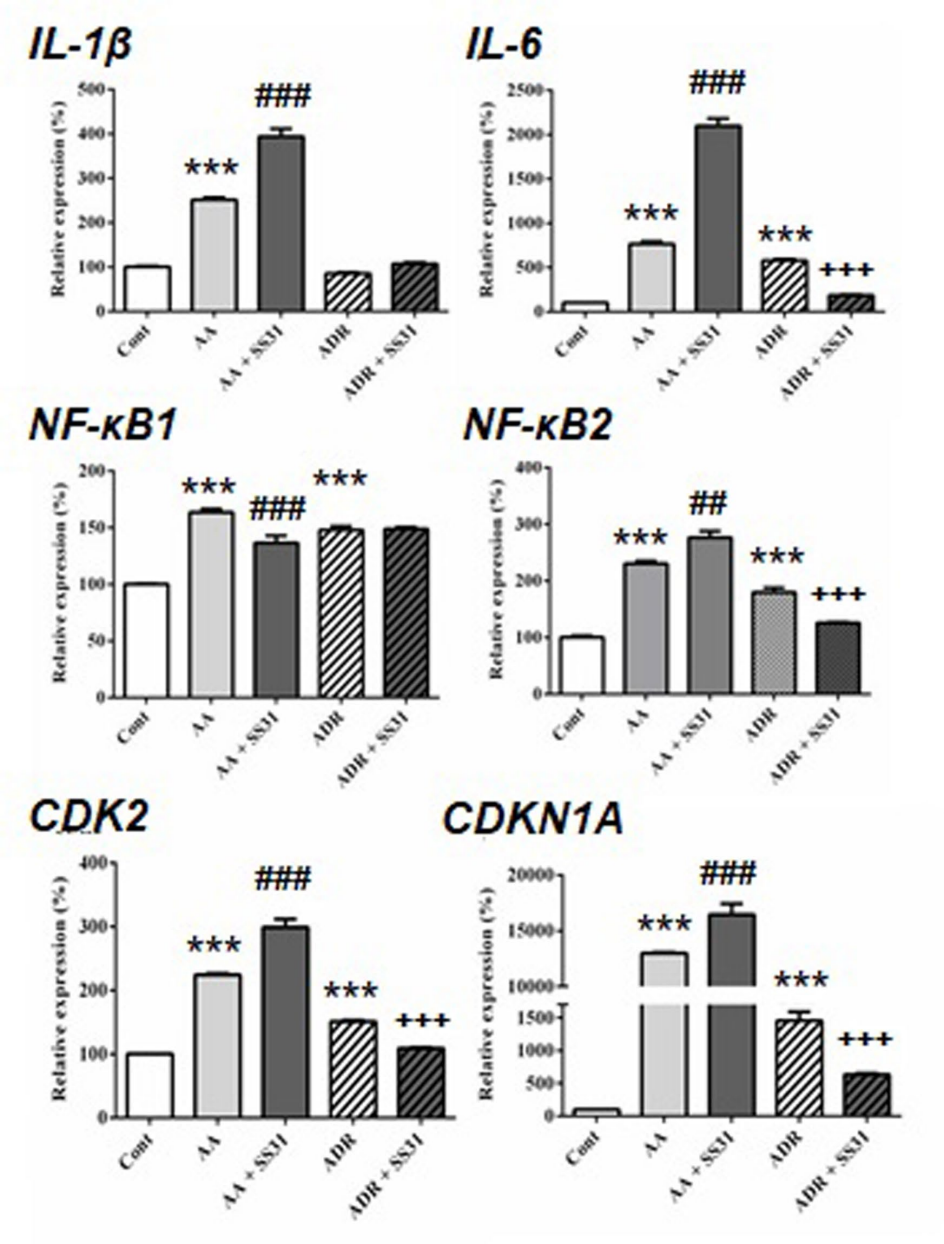
Expression of acute pro-inflammatory pathway-responsive genes in the kidneys of mice exposed to aristolochic acid or adriamycin, without or with SS-31. After 7 days of administration of either aristolochic acid (AA) or adriamycin (ADR), alone or together with SS-31 (+SS31), the animals (n = 5 mice per experimental group) were sacrificed and their kidneys retrieved for the determination by qRT-PCR of the mRNA levels of genes of components of acute inflammatory pathways. Healthy wild-type BALB/c male mice were used as controls (Cont). The levels of expression of the mRNAs for interleukin (IL)-β, IL-6, nuclear factor kappa-light-chain-enhancer of activated B cells (NF-κB)1, NF-κB2 and cyclin-dependent kinases (CDK2 and CDKN1A) were quantified by qRT-PCR. Results were averaged for all animals in each experimental group and are presented as % of changes in the mRNA levels in the treated animals *versus* control animals ± sem.*: AA or ADR compared to Cont; ^#^: AA + SS-31 or ^+^: ADR + SS-31 compared to AA or ADR alone, respectively. (*p < 0.05; **p < 0.01; ***p < 0.001; applies to all symbols).

One week after the onset of disease, control wild-type mice, AA- or ADR-administered mice, and SS-31-treated mice were sacrificed, and the kidneys were retrieved for the determination by qRT-PCR of markers of oxidative stress (**Figure 2**), inflammation, and cell proliferation (**Figure 3**). The regulatory effect of SS-31 on enzymes associated with oxidative stress, including hypoxia-inducible factor 1α (HIF-1α), cyclooxygenase-2 (COX-2), and heme oxygenase-1 (HO-1), was model dependent (**Figure 2**). In both experimental models, mRNA levels of *HIF-1α*, *HO-1*, and *COX-2* were significantly increased, and SS-31 could restore these levels only for *HIF-1α* and *HO-1 *(as compared to control mice),in AA- and ADR-administered mice, respectively. Surprisingly, treatment with SS-31 further increased the expression of *COX-2* in acutely damaged kidneys. In our experimental setting, the expression of superoxide dismutase-1 (*SOD1*) at the mRNA level was not significantly affected during acute renal injury, while *SOD2* expression was downregulated and partially restored after SS-31 treatment in the AA model. Attempts to directly measure ROS production in kidney sections were not successful using our experimental models.

We next evaluated the expression of genes involved in acute inflammatory pathways and the effect of SS-31 in kidneys of mice exposed to AA and ADR (**Figure 3**). Except for the expression of the transcription factor nuclear factor kappa-light-chain-enhancer of activated B cells (NF-κB)1, SS-31 treatment did not reduce the expression of inflammatory genes in the AA model, possibly because of the severity of induced kidney lesions as seen in the histological slides (**Figure 1**). In the ADR model, SS-31 treatment could regulate the expression of the pro-inflammatory cytokine interleukin (IL)-6 and NF-κB2 at the mRNA level. Finally, we analyzed the expression of cell proliferation markers in our experimental setting and the effect of SS-31 treatment. Similar to early inflammatory pathway-associated genes, SS-31 treatment could not modulate the upregulation of cyclin-dependent kinase 2 (CDK2), an enzyme involved in the G1-S transition phase of the cell cycle, induced during severe acute kidney injury after AA administration. SS-31 could, however, regulate the expression of this gene following ADR administration, which resulted in less acutely severe inflammation (IL-1β, IL-6) and renal damage on histology.

### Effects of SS-31 on Components of the RAS

**Figure 4:**
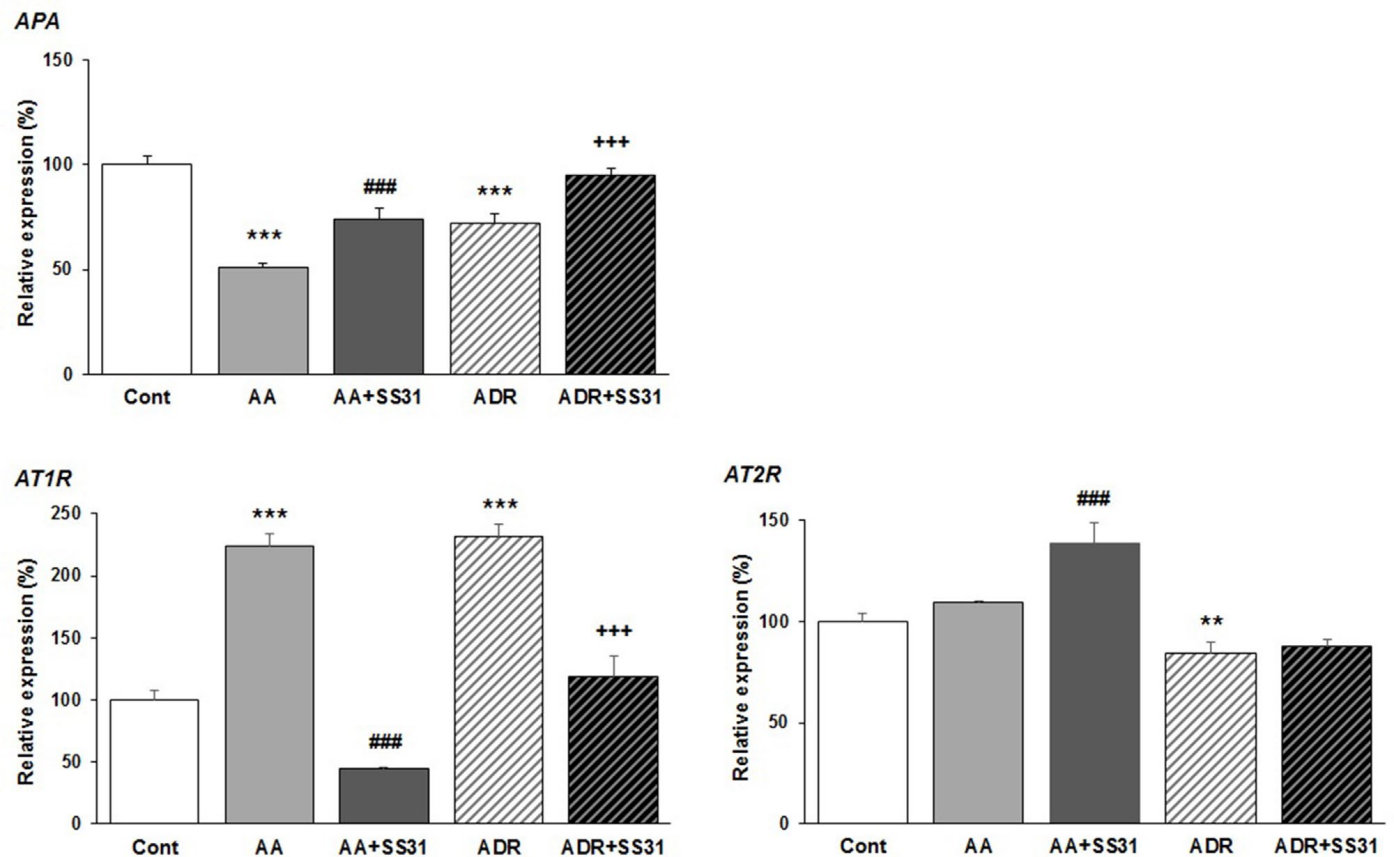
Modulation of genes of the renin–angiotensin system in the kidneys of mice exposed to aristolochic acid or adriamycin, without or with SS-31. After 7 days of treatment with either aristolochic acid (AA) or adriamycin (ADR), alone or together with SS-31 (+SS31), the animals (n = 5 mice per experimental group) were sacrificed and their kidneys retrieved for the determination by qRT-PCR of the mRNA levels of genes of the components of the renin–angiotensin system (RAS). Healthy wild-type BALB/c male mice were used as controls (Cont). The levels of expression of the mRNAs for aminopeptidase A (APA), the AT_1_ and AT_2_ angiotensin receptors were quantified by qRT-PCR. Results were averaged for all animals in each experimental group and are presented as % of changes in the mRNA levels in the treated animals *versus* control animals ± sem.*: AA or ADR compared to Cont; ^#^: AA + SS-31 or ^+^: ADR + SS-31 compared to AA or ADR alone, respectively. (*p < 0.05; **p < 0.01; ***p < 0.001; applies to all symbols).

Beyond the hemodynamic effects of the RAS in the kidney, local activation of this pathway has been associated with renal damage, and as such, RAS blockers are often administered to patients with chronic kidney disease (Kobori et al., 2007; Urushihara and Kagami, 2017). Overactivation of the angiotensin type 1 receptor (AT1R) by the octapeptide Ang II promotes the development and progression of several kidney diseases* via *signaling cascades involving inflammation and fibrosis, whereas the activation of AT_2_R may counterbalance AT_1_R activation, thus being beneficial. Conversion of Ang II to Ang III is critical for AT_2_R-mediated effects in the kidney since Ang III is the major agonist of AT_2_R (Padia et al., 2008). Ang III is released from Ang II by the action of APA removing the N-terminal Asp of Ang II. To evaluate the effect of SS-31 on the RAS, we therefore determined the expression of AT_1_R, AT_2_R, and APA in the kidneys of mice exposed to either AA or ADR, without or with SS-31. Following both tubulo-interstitial or glomerular damage induced by AA or ADR administration to the mice, significant downregulation of the expression of APA mRNA was observed by qRT-PCR (**Figure 4**) in renal tissues, which was nearly restored by concomitant SS-31 treatment. Accordingly, AT_1_R mRNA levels, which were upregulated during acute kidney injury mediated by AA or ADR, decreased upon SS-31 treatment. Interestingly, at least in the AA group, SS-31 also upregulated the expression of AT_2_R mRNA. Overall, in addition to acute oxidative stress, inflammatory and proliferative pathways induced locally during renal tissue injury, SS-31 could modulate the RAS, notably by regulating the expression of APA and consequently of AT_2_R.

**Figure 5:**
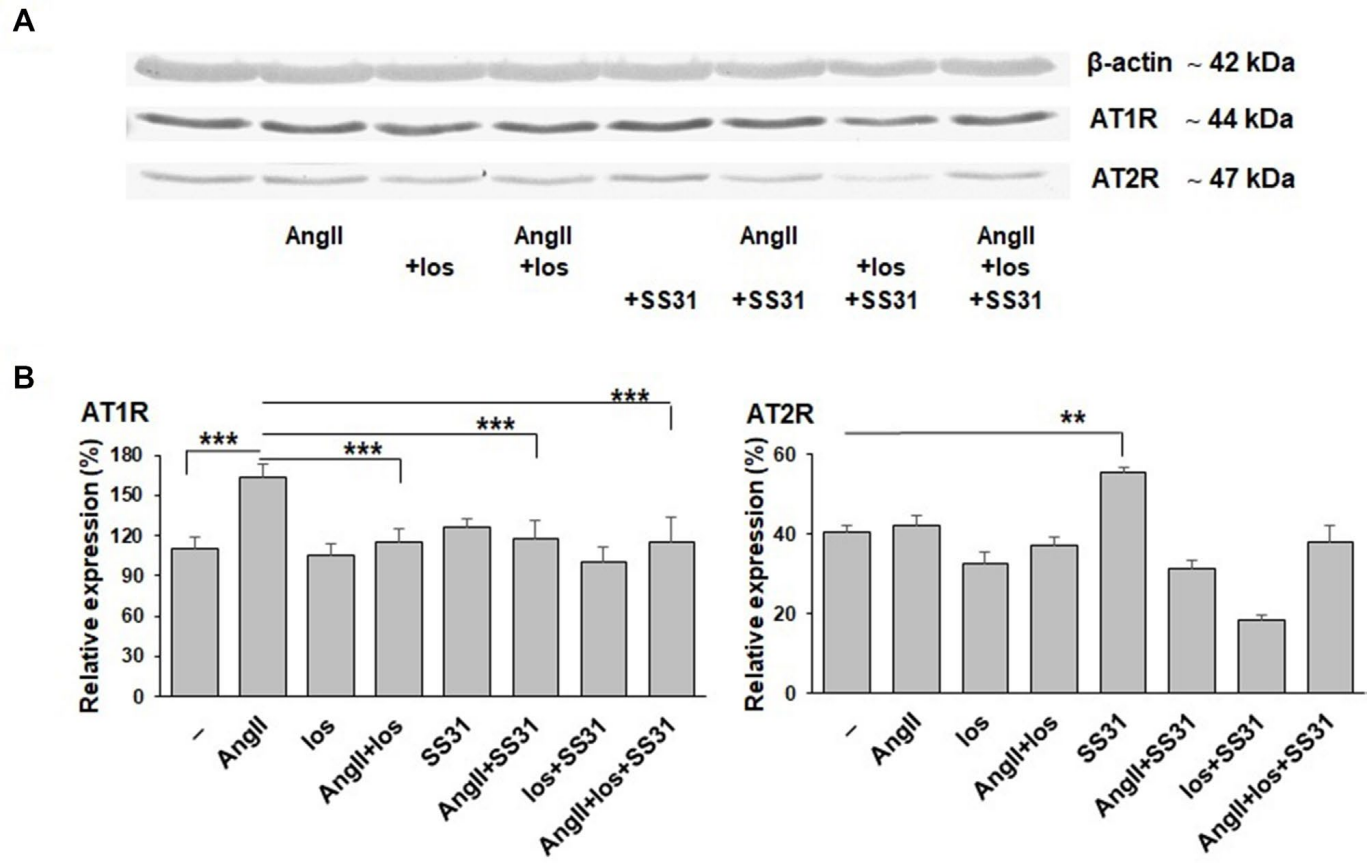
Expression of AT_1_R and AT_2_R proteins by EC219 cells exposed to Ang II, an AT_1_R antagonist (losartan) and SS-31. The expression of AT_1_R and AT_2_R by EC219 cells exposed for 24 h to either Ang II, the AT_1_R antagonist losartan or SS-31, or their combination, were analyzed by western blotting: (one representative blot, **(A)**, then quantified (3 experiments, **(B)** by comparison with the expression of β-actin as a control. (**p < 0.01; ***p < 0.001).

We next analyzed whether SS-31 could directly regulate the expression of AT_1_R and AT_2_R at the protein level. For this purpose, we used an *in vitro* cellular model that expresses these receptors (as previously characterized and published, Juillerat-Jeanneret et al., 1992), exposed to Ang II, losartan (a specific AT_1_R antagonist used in the clinic), SS-31, or their combination (**Figure 5**). SS-31 was able to increase the expression of AT_2_R and in combination with losartan to control AT_1_R upregulation. Therefore, these protein data support the *in vivo* mRNA expression levels showing the regulation by SS-31 of the RAS in acutely injured mouse renal tissues, including the expression of AT_2_R. This information suggests that SS-31, alone or in combination with RAS blockers, may be of clinical interest for developing nephroprotective therapies.

### Design and *ex Vivo* and *in Vitro* Evaluation of Functionalized Analogs of SS-31

**Figure 6:**
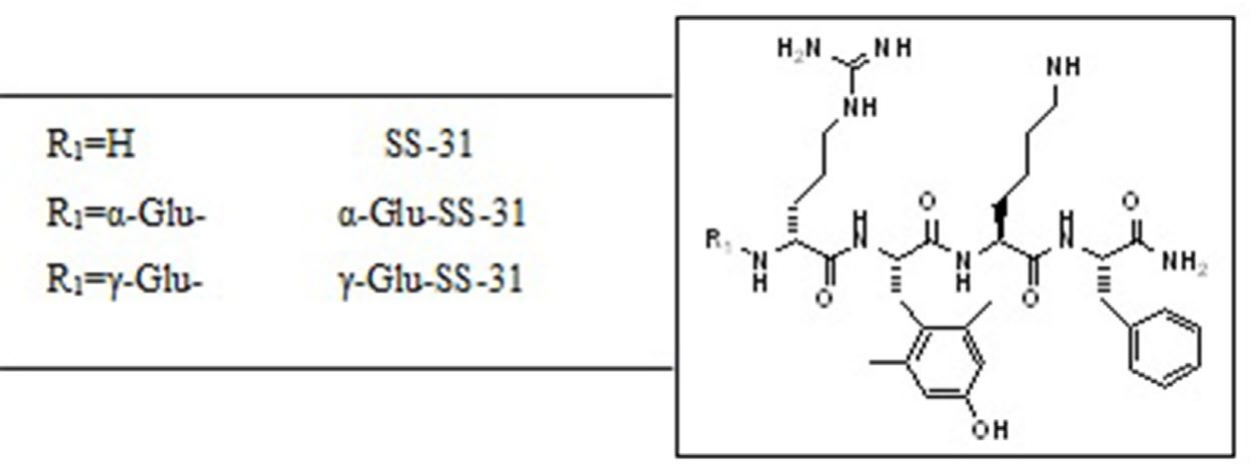
Chemical structures of SS-31 and the evaluated functionalized analogs. The prodrugs are composed of the active compound (SS-31) and the targeting α-Glu or γ-Glu-moiety as substrates for the enzymes aminopeptidase A (APA) and γ-Glu-transpeptidase (γ-GT), respectively.

As shown in **Table 1**, similar amounts of SS-31 were found in the plasma, liver, and kidneys of wild-type mice after i.p. injection, which could result in generalized off-target effects during therapeutic administration. Thus, enhancing the biodistribution of this peptide selectively to the kidney, in particular to the diseased renal tissue, may be a valuable option to enhance therapeutic efficacy while decreasing exposure of non-diseased organs. We had previously observed highly increased enzymatic activities of the peptidases APA and γ-GT in selective compartments of diseased kidneys in rodent experimental models as well as in human samples (Juillerat-Jeanneret et al., 2015). We therefore designed prodrugs of SS-31 as substrates for these enzymes by adding either α-Glu (as a substrate for APA) or γ-Glu (as a substrate for γ-GT) at the N-terminus of SS-31. The chemical structures of the compounds are shown in **Figure 6**.

**Figure 7:**
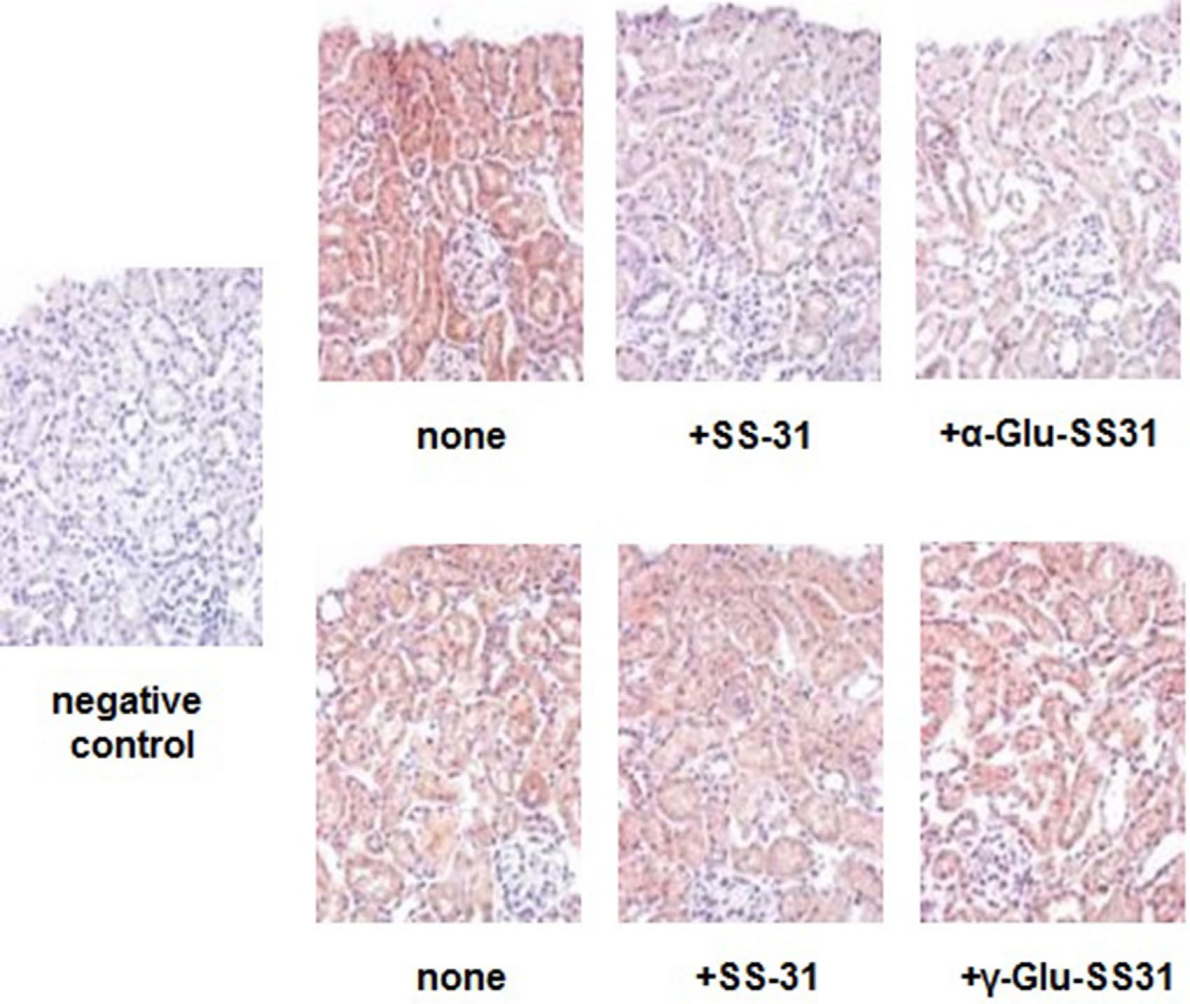
Selective inhibition of the enzymatic activities of aminopeptidase A and γ-glutamyltranspeptidase by SS-31, α-Glu-SS-31 or γ-Glu-SS-31 determined by histoenzymography in the kidneys of mice exposed to adriamycin. Sequential sections (7 µm) of frozen OCT-embedded kidneys of adriamycin (ADR)-treated mice were exposed at 37°C to β-methoxynaphthylamide (β-NA) specific substrates of aminopeptidase A (α-Glu-βNA, upper panels) or γ-glutamyltranspeptidase (γ-Glu-βNA, lower panels) and Fast Blue B, in the absence (none) or the presence of SS-31, α-Glu-SS-31 or γ-Glu-SS-31. The sections were then counterstained with light hematoxylin reagent. The enzymatic activity is visualized as a red precipitate. For the negative control, we used Fast Blue B in the absence of β-NA substrate. Representative renal cortical sections per experimental groups are shown (200x magnification).

First, we evaluated the effects of SS-31, α-Glu-SS-31, and γ-Glu-SS-31 on the activities of APA and γ-GT using an *ex vivo* approach by histoenzymography on histological slides of frozen kidney samples of mice exposed to ADR for 7 days (**Figure 7**). For these proof-of-concept assays, we limited our experiments to the ADR model in which kidney structures are better preserved because of less severe acute lesions. Using this model, we confirmed our previous data showing the increased enzymatic activity of the enzymes APA and γ-GT in the kidneys of ADR-exposed mice when compared to wild-type mice, mainly in the glomerulus and proximal tubular cells (results not shown; Juillerat-Jeanneret et al., 2015). The enzymatic activity of APA was selectively inhibited by SS-31 and α-Glu-SS-31, its cognate substrate, but not γ-Glu-SS-31 (not shown), whereas the activity of γ-GT was modified neither by SS-31 or by its cognate substrate γ-Glu-SS-31, suggesting a more interesting potential of APA for developing substrate prodrugs.

**Figure 8:**
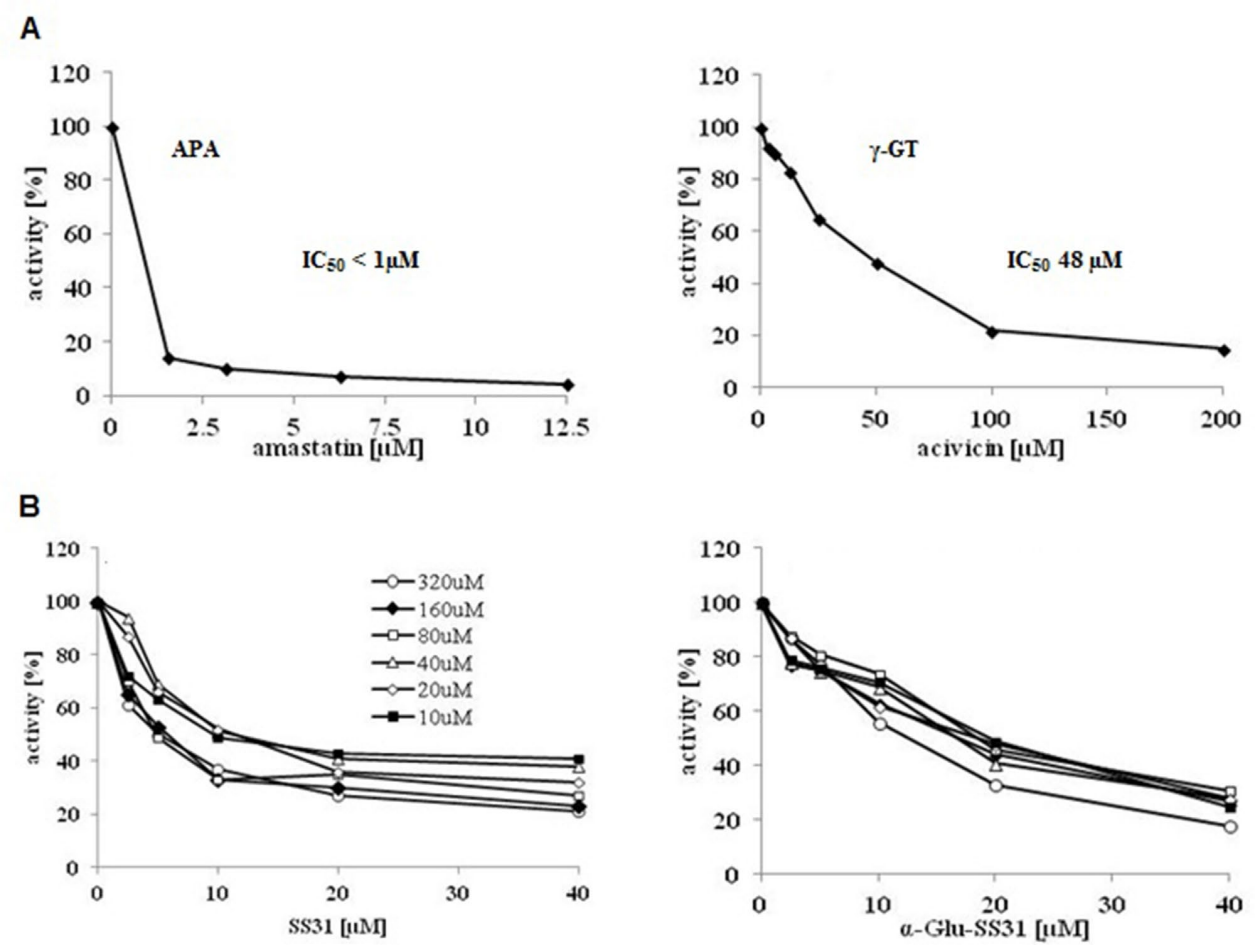
Selective inhibition of the enzymatic activity of aminopeptidase A by SS-31 and its prodrug α-Glu-SS-31 in living EC219 cells. **(A)** Intact living EC219 cells were exposed to increasing concentrations of either amastatin (left panel) or acivicin (right panel), two well-characterized enzymatic activity inhibitors of APA or γ-GT, respectively. The activities of the enzymes aminopeptidase A (APA) or γ-glutamyltranspeptidase (γ-GT) were determined using 40 µM of the substrates (S), α-Glu-AMC or γ-Glu-AMC, respectively. **(B)** Intact living EC219 cells were exposed to increasing concentrations [10-320 µM] of the APA α-Glu-AMC substrate, in the presence of increasing concentrations of SS-31 or α-Glu-SS-31, and the increase of free AMC fluorescence was recorded. Data were analyzed graphically. (DMSO 0.2% was present in all samples).

In order to analyze the effects of SS-31 and its selective analogs, previously characterized renal epithelial cell lines and non-renal cells were screened to determine their levels of APA and γ-GT enzymatic activities (**Supplementary Table S2**). In comparison to the available renal epithelial cell lines, the EC219 rat endothelial cells expressed very high levels of both enzymatic activities (Juillerat-Jeanneret et al., 1992). We therefore used intact living EC219 cells to compare the inhibitory effects of SS-31 and α-Glu-SS-31 on APA enzymatic activity (**Figure 8** **)**. To validate the inhibition methodology, we determined as controls the inhibition of APA by amastatin and of γ-GT by acivicin, two validated synthetic inhibitors of these enzymes. DMSO used as solvent for the different drugs had only very limited effect of the activities of the enzymes (data not shown). We observed the selective inhibition of APA activity in EC219 cells by SS-31 and α-Glu-SS-31, its cognate substrate, but not γ-Glu-SS-31 (data not shown), while neither SS-31 nor α-Glu-SS-31 was able to inhibit the activity of γ-GT in intact EC219 cells (data not shown). The IC_50_ values for APA inhibition were determined to be 8.5 ± 3.8 µM for SS-31 and 13.7 ± 6.7 µM for α-Glu-SS-31. Thus, both SS-31 and α-Glu-SS-31 displayed comparable inhibitory potential of APA activity, with IC_50_ being only very slightly lower for free SS-31.

Finally, we determined if α-Glu functionalization of SS-31 would improve the plasma stability of the compound. SS-31 or α-Glu-SS-31 (at 1 µM concentration) was added to heparinized mouse plasma, and the aliquots were incubated at 37°C for various time points before analysis (**Table 2**). After a rapid decrease of α-Glu-SS-31 concentration, associated with the appearance of free SS-31, in mouse plasma, the concentration of both compounds remained stable for up to 5 h, whereas SS-31 concentration decreased regularly during the same period of time.

## Discussion

Excessive ROS production in response to acute inflammatory disorders or toxic injuries causes oxidative damage to lipids, nucleic acids, and proteins, leading to tissue dysfunction, whereas physiological production of ROS is critical for cell signaling and homeostasis. Therefore, ROS levels are tightly regulated by complex cellular anti-oxidative systems. ROS are mainly the products of mitochondrial oxidative phosphorylation. Mitochondrial changes are a feature of kidney diseases, suggesting that reducing ROS generation may be of therapeutic interest. Several therapeutics, including DHL-HisZn, a lipoic acid derivative; probucol, an anti-hyperlipidemic drug with ROS scavenging properties; or metformin, an anti-diabetic, were shown to have anti-oxidative properties and to enhance renal recovery after ischemia–reperfusion injury (Morales et al., 2010; Koga et al., 2012; Zhou et al., 2013). Antioxidant tetrapeptides based on the YRFK (Tyr-Arg/D-Arg-Phe-Lys) peptide sequences, conjugated to a triphenylphosphonium moiety to improve pharmaco-chemical stability, have been designed (Akhmadishina et al., 2018). Other mitochondrial-penetrating peptide sequences based on cyclohexylAla and D-Arg have been evaluated (Hidaka et al., 2017). An interesting molecule in this context is the mitochondria-targeting antioxidant tetrapeptide SS-31 (D-Arg-2,6-dimethyltyrosine-Lys-Phe-NH_2_, Bendavia). SS-31 belongs to a family of aromatic cationic peptides that selectively target the mitochondrial inner membrane and can scavenge ROS, an effect mediated by the dimethyl-Tyr group of the peptide (Zhao et al., 2004; Dai et al., 2011) reducing mitochondrial production of ROS. SS-31 has been shown to improve the course of diverse experimental models of kidney diseases associated with mitochondrial oxidative stress (Szeto et al., 2011; Zhao et al., 2013; Tabara et al., 2014; Szeto et al., 2016; Sweetwyne et al., 2017; Danielle et al., 2018). SS-31 was also shown to improve pulmonary hypertension in murine models of the disease (Lu et al., 2015), atherosclerotic plaques in mice in part by increasing SOD activity (Zhang et al., 2017), or oxidative stress in cells from patients with Friedreich ataxia, a progressive neurodegenerative disease (Zhao et al., 2017).

To investigate potential beneficial nephroprotective effects of SS-31 other than direct anti-oxidant properties, we developed two *in vivo* murine models of acute tubular and glomerular damage mediated by toxic compounds. AAs are a family of 10-nitro-1-phenantropic acids derived from tyrosine. AA is a natural herbal compound that is toxic to the renal tubulo-interstitial compartment, leading to a rapidly progressive nephropathy, renal failure, and, in some instances, cancer of the urinary tract. AA-induced tubular toxicity involves mitochondrial permeability and injury, leading to defective activation of anti-oxidative enzymes, combined with progressive tubular atrophy, impaired regeneration, and apoptosis/autophagy of proximal tubular epithelial cells (Romanov et al., 2011; Zeng et al., 2014). The anticancer drug doxorubicin (ADR) and its metabolites accumulate in the inner mitochondrial membrane, producing excessive ROS and causing oxidative damage. ADR administration results in a more progressive kidney disease characterized by massive proteinuria due to podocyte injury, glomerulosclerosis, tubulo-interstitial inflammation, and fibrosis (Okuda et al., 1986; Mukhopadhyay et al., 2009; Lee and Harris, 2011; Gao et al., 2014). Cardiolipin, a lipid of interest for SS-31 functions, was shown to interact with doxorubicin (Aryal and Rao, 2016). In the present report, we showed that SS-31 could alleviate the severity of nephropathy in mice exposed to AA or ADR. We also showed that SS-31 reduced the upregulation at the mRNA levels of the stress response-associated NF-κB signaling pathway. SS-31 also modulated the expression of genes involved in hypoxia and oxidative stress (mainly *HIF-1α* in the AA and *HO-1* in the ADR model), in our experimental setting. Thus, using different models of acute nephropathy, our results extend and confirm previous experimental data showing the protective role of SS-31 against acute oxidative stress injuries and inflammation in the kidney and other organs (Mizuguchi et al., 2008; Szeto et al., 2011; Huang et al., 2013).

Interestingly and unexpectedly, we observed that, beyond the regulation of acute oxidative stress and associated inflammatory responses, SS-31 could directly modulate the expression of members of the RAS, in particular APA and AT_2_R. The RAS is a main regulator of kidney functions, being modulated and acting independently in the blood and the kidney (Sparks et al., 2014). All the RAS components have been found in the kidney, with AT_1_R and AT_2_R being differentially expressed in renal tissues. Ang II* via *AT_1_R mediates most of the classical functions of the RAS. Non-hemodynamic actions of AT_1_R include enhanced generation of ROS, oxidative stress, and inflammatory responses. In cardiomyocytes and podocytes, Ang II/AT_1_R activation can induce apoptosis mediated by mitochondrial fission (Qi et al., 2018). AT_2_R counteracts AT_1_R effects, with AT_2_R being abundant during fetal development but also detectable in adult tissues, including the kidney (Mukoyama et al., 1993). In the adult kidney, AT_2_R is expressed by glomerular, vascular and tubular cells (particularly highly expressed in proximal tubules). AT_2_R stimulation can inhibit inflammation involving NF-κB, reduce organ damage, and promote tissue repair and regeneration. The effects of the AT_2_R agonists C21 or NP-6A4 (Toedebush et al., 2018) suggest that AT_2_R activation may limit renal damage, in part mediated by improved function of mitochondria. Thus, AT_1_R and AT_2_R display opposing functions and selectivity (Carey and Padia, 2013). In the intact kidney, the Ang II/AT_1_R axis is predominant in response to initial injury, stimulating oxidases to produce ROS, inducing oxidative and inflammatory stresses. The Ang II/Ang III/AT_2_R axis becomes apparent following pathological conditions, when ongoing local injury increases AT_2_R expression. APA is a membrane-bound zinc-dependent aminopeptidase broadly expressed in the kidney (located at the surface of glomerular vascular and mesangial cells as well as of podocytes and proximal tubules). Conversion of Ang II to Ang III (desAsp^1^-Ang II) by APA is critical for AT_2_R-mediated effects in the kidney since Ang III is the major agonist of AT_2_R (Wolf et al., 1997; Padia et al., 2008; Kemp et al., 2012). SS-31 displays an N-*term-*Arg, as does Ang III, which stabilizes the binding of Ang III to AT_2_R. This may represent a physiological response to kidney stresses, including oxidative stress. We have previously shown that transforming growth factor β can regulate APA expression (Juillerat-Jeanneret et al., 2000). Similarly, others have shown a role for several cytokines and growth factors (Suganuma et al., 2004), linking APA regulation to inflammatory pathways. As pharmacological interventions for the treatment of many nephropathies involve agents that block the RAS (Thomas and Groop, 2011; Lambers Heerspink and de Zeeuw, 2013), our observations of a link between SS-31, the RAS, and inflammatory/oxidative stress reactions have implications for potential therapeutic effects of SS-31 besides its direct anti-oxidative properties.

As ROS also play important roles in normal physiology, chronic inhibition of ROS may result in off-target responses and side effects. Previous attempts have been done to increase mitochondria targeting of therapeutic drugs, mostly using conjugation of agents to various compounds (Wolf et al., 1997, Wolf et al., 2000), including nanoparticles (Hidaka et al., 2017; Akhmadishina et al., 2018; Battogtokh et al., 2018; Mattarei et al., 2018; Shi et al., 2018). We have previously shown that high enzymatic activities of the peptidases APA and γ-GT in diseased human and rat kidneys may represent targets to develop prodrug therapeutics (Juillerat-Jeanneret et al., 1992; Juillerat-Jeanneret et al., 2015). In our murine models, APA activity was expressed in the glomerular tuft and proximal tubular cells, while γ-GT activity was confined to proximal tubular epithelial cells. Using similar experimental models. we have previously developed prodrugs of γ-secretase inhibitors (GSI) as substrates for peptidases to control pathogenic Notch pathway activation. In this previous research, we demonstrated that targeting γ-GT activity using a γ-Glu-linker-GSI was the preferred choice as compared to targeting APA activity (Wyss et al., 2018). For the present project, we directly bound α-Glu or γ-Glu to SS-31 using a peptide bond as functionalized prodrugs of SS-31, designed as substrates of these two peptidases. Whereas we previously showed that to achieve the targeting of prodrug inhibitors of the membrane-inserted γ-secretase was more efficient using substrates for γ-GT, we show here that for targeting SS-31 and mitochondrial pathways, the use of prodrug substrates for APA is to be preferred. In our experimental setting, SS-31 acted as an inhibitor of APA enzymatic activity, resulting in a slight upregulation of APA mRNA levels, likely as a feedback mechanism. Several inhibitors for this enzyme have been developed as potentially therapeutic (Mentzel et al., 1996; Mentzel et al., 1997; David-Basei et al., 2001; Gerlofs-Nijland et al., 2001; Chen et al., 2014). Thus, in addition to SS-31, such inhibitors associated with AT_2_R modulators may be of value in the context of kidney diseases.

## Data Availability Statement

The datasets generated for this study are available on request to the corresponding author.

## Ethics Statement

The animal study was reviewed and approved by the animal ethics committee of the Canton de Vaud, Switzerland (No 2655.0).

## Author Contributions

LJ-J and DG conceived and administered the project, analyzed the results, and wrote the manuscript. JA participated in the design of the project, designed and prepared the compounds, and participated in the evaluation of the results and the writing of the manuscript. J-CW, RK, JM, MS, J-LM, and DG performed the experiments and participated in the analysis of the results and the writing of the manuscript.

## Funding

This work was financially supported by the Roche Postdoc Fellowship program, DG was supported by Fondation Pierre Mercier pour la Science and Fondation Medi-CAL Futur. The authors declare that this study received funding from F. Hoffmann–La Roche. The funder had the following involvement with the study: synthesis of SS-31 and prodrug analogs, as well as HPLC procedures for pharmacokinetic studies. The funders had no role in study design, data collection and analysis, decision to publish, or preparation of the manuscript.

## Conflict of Interest

MS, J-LM, and JA are employees of F. Hoffmann–La Roche but declare no conflict of interest. The remaining authors declare that the research was conducted in the absence of any commercial or financial relationships that could be construed as a potential conflict of interest.
