# Supplementary material for: Differential Effects of the Mitochondria-Active Tetrapeptide SS-31 (D-Arg-dimethylTyr-Lys-Phe-NH2) and Its Peptidase-Targeted Prodrugs in Experimental Acute Kidney Injury
Source: Front Pharmacol. 2019 Nov 8;10:1209. doi: 10.3389/fphar.2019.01209 (PMC6857474; doi:10.3389/fphar.2019.01209)
Supplement: Supplementary file 1 [file DataSheet_1.docx]

**Differential effects of the mitochondria-active tetrapeptide SS-31 (D-Arg-dimethylTyr-Lys-Phe-NH_2_) and its peptidase-targeted prodrugs in experimental acute kidney injury**

*Jean-Christophe Wyss^1^, Rajesh Kumar^1^, Josip Mikulic^1^, Manfred Schneider^2^, Jean-Luc Mary^2^, Johannes D. Aebi^2^, Lucienne Juillerat-Jeanneret^1,3^ and Dela Golshayan^1*^.*

**Supplementary tables**

**Table S1**. **Sequences of the primers used for the qPCR experiments**

| ***Gene symbol*** | ***Forward Sequence (5'–3')*** | ***Reverse Sequence (5'–3')*** |
| --- | --- | --- |
| *APA* | TGGACTCCAAAGCTGATCCT | TCAGCCCATCTGACTGGAAT |
| *AT_1_R* | ACTCACAGCAACCCTCCAAG | CTCAGACACTGTTCAAAATGCAC |
| *AT_2_R* | GGAGCTCGGAACTGAAAGC | CTGCAGCAACTCCAAATTCTT |
| *CDK2* | TTCCTCTTCCCCTCATCAAG | ACGGTGAGAATGGCAGAAAG |
| *COX-2* | CAGGTCATTGGTGGAGAGGTG | TGCTCATCACCCCACTCAGG |
| *GAPDH* | GTCGGTGTGAACGGATTTG | AAGATGGTGATGGGCTTCC |
| *HIF-1α* | TCACCAGACAGAGCAGGAAA | GTCACCTGGTTGCTGCAATA |
| *HO-1* | CGCCTTCCTGCTCAACATT | TGTGTTCCTCTGTCAGCATCAC |
| *IL-1β* | GGGCCTCAAAGGAAAGAATC | CTCTGCTTGTGAGGTGCTGA |
| *IL-6* | AGAAGGGCCTGGAATGAAAC | AAGACCCTGCTGGAACAAGA |
| *NF-κB1* | GGGTCTGGGGATACTGAACA | GCCTCCATCAGCTCTTTGAT |
| *NF-κB2* | TGGAACAGCCCAAACAGC | CACCTGGCAAACCTCCAT |
| *SOD1* | CAGGACCTCATTTTAATCCTCAC | TGCCCAGGTCTCCAACAT |
| *SOD2* | TGGACAAACCTGAGCCCTAA | GACCCAAAGTCACGCTTGATA |

**Table S2. Characteristics of the cell lines evaluated**

The evaluation of APA and γ-GT enzymatic activities in the different cell lines was performed in intact living cells exposed to the fluorigenic APA and γ-GT specific peptidyl-AMC substrates.

**Cell line Description Origin Species  *Enzymatic activities***

***APA γ-GT***

_______________________________________________________________________

MDCK kidney epithelium distal tubule canine + +/-

LLCPK kidney epithelium proximal tubule pig - +/-

mDCT kidney epithelium distal tubule mouse ++ ++

mCCD kidney epithelium collecting tubule mouse + +

EC219 brain endothelium microvascular rat +++ +++

_____________________________________________________________________

-: no measurable activity; +/-: very low activity; +: low activity; ++: medium activity; +++: high activity.
